# The Chemokine CXCL16 is Highly and Constitutively Expressed by Human Bronchial Epithelial Cells

**DOI:** 10.1080/01902140802635517

**Published:** 2009-05-04

**Authors:** Caroline Day, Rashmika Patel, Cristina Guillen, Andrew J. Wardlaw

**Affiliations:** Institute for Lung Health, Department of Infection, Immunity and Inflammation, Glenfield Hospital, University of Leicester, Leicester, UK

**Keywords:** bronchial epithelium, chemokine, CXCL16, CXCR6, T-cell homing

## Abstract

The chemokine receptor CXCR6 is highly expressed on lung-derived T cells compared to blood T cells, especially in inflammatory diseases characterised by T-cell migration to the lung. This suggests that CXCR6 is a candidate lung homing receptor. The sole ligand of CXCR6, CXCL16, has previously been shown to be expressed by alveolar macrophages. The authors hypothesized that also structural lung cells express CXCL16. CXCL16 expression was detected using real-time reverse transcriptase–polymerase chain reaction (RT-PCR), Western blotting, enzyme-linked immunosorbent assay (ELISA), and flow cytometry. Chemotaxis assays were used to test functionality of the secreted protein. Human bronchial epithelial cells secreted relatively high basal levels of CXCL16 (> 1000 pg/mL). Interferon (IFN)-γ, but not tumor necrosis factor (TNF)-α or interleukin (IL)-4, caused a modest but significant up-regulation in secretion. Airway smooth muscle and fibroblasts also expressed CXCL16, but at lower levels. Western blotting detected expression of the full-length (60-kDa) form of the chemokine in cell lysates, and the cleaved (35-kDa) form in culture supernatants. Concentrated supernatants from a bronchial epithelial cell line (BEAS-2B) were chemotactic for CXCR6 expressing T cells from blood. In conclusion, these results suggest that the bronchial epithelium is an important source of constitutively expressed CXCL16, which may be involved in T-cell recruitment to the lung in health and disease.

The lung is constantly exposed to airborne antigens, but despite the considerable quantities of foreign particles inhaled, the lung is in the healthy individual a quiescent organ. However, in conditions such as asthma or sarcoidosis, T cells are thought to be involved in an inappropriate immune response causing chronic pulmonary inflammation [1, 2]. Therefore, investigating the mechanism by which T cells are recruited to the lung is important, because targeting components involved in this pathway could be of therapeutic interest.

It is not completely understood how lymphocyte recruitment to and microcompartmentalization within the lung is regulated, but chemokines and their receptors are likely to be central to this process. Memory T cells preferentially return to the organ that was drained by the lymph node where they were sensitized in a process known as homing. This strictly controlled migration is in part regulated by chemokines [3]. More than 50 human chemokines have been identified that bind to and activate over 20 different 7-transmembrane–spanning, G protein–linked chemokine receptors [4]. The chemokines involved in homing to the skin and gut are well defined, but it is not known whether there is specific homing to the lung [5, 6]. We and others have shown that the chemokine receptor, CXCR6, is highly expressed on lung derived T cells compared to blood T cells, especially in inflammatory diseases characterized by T-cell infiltration in the lung, such as sarcoidosis, suggesting that CXCR6 is a candidate lung homing receptor [7, 8]. The two initial studies of the only ligand of CXCR6, CXCL16, reported the lung as having high levels of mRNA expression of this chemokine [9, 10]. We have detected relatively high concentrations of CXCL16 in bronchoalveolar lavage compared to other chemokines from both normal subjects and patients with asthma and sarcoidosis, which implies a constitutive expression of CXCL16 by cells in the lung [7]. We also found that alveolar macrophages produce substantial amounts of CXCL16 in vitro [7]. We were interested in investigating if other cell types in the lung, particularly bronchial epithelial cells, express CXCL16, and thereby contribute to the recruitment of T cells to the lung. The bronchial epithelium, which provides a barrier between the lumen and the submucosa, is actively involved in both innate and acquired immune response and in the pathogenesis of airway inflammation [11]. The epithelium is also known to be an important source of chemokines in other organs such as skin and gut and is responsible for the infiltration of lymphocytes to these sites [5, 6]. We hypothesized that the airway epithelium produces CXCL16, playing an essential role in the specific migration of T cells to the lung. CXCL16, together with CX_3_CL (fractalkine), forms the small group of chemokines that exists in a transmembrane form as well as a soluble form. Whereas the soluble form has chemotactic activity, [9] the membrane-bound form functions as a cell adhesion molecule, a scavenger, and a phagocytic receptor for oxidized low-density lipoproteins and bacteria [12, 13]. Shedding of the membrane-bound form is believed to occur through the action of a disintegrin and metalloprotease (ADAM)-10, which cleaves the protein into the soluble form [14, 15]. CXCL16 has been shown to be involved in the recruitment of T cells to inflamed liver and the joints of patients with rheumatoid arthritis, but there has been no reports suggesting CXCL16 expression in these organs in the absence of inflammation [16, 17].

In this work, we have characterized the CXCL16 expression of a human bronchial epithelial cell line (BEAS-2B) and assessed the functionality of the secreted protein in a chemotaxis assay, where T cells from blood migrated to BEAS-2B culture supernatants. We have also confirmed expression of CXCL16 in cultures of primary human bronchial epithelial cells, fibroblasts, and smooth muscle cells and studied the effect of interferon-gamma (IFN-γ) on CXCL16 expression by these cells.

## MATERIALS AND METHODS

### Cells

An simian virus 40 (SV40)-transformed human bronchial epithelial cell line, BEAS-2B, was purchased from European Collection of Cell Cultures (Salisbury, Wiltshire, UK) and cultured in bronchial epithelial growth medium (TCS Cellworks, Buckingham, UK) on fibronectin-coated plates (0.4 mg/mL; Sigma, Dorset, UK). Primary human epithelial cells were acquired from bronchial brushings from asthmatic or nonasthmatic patients and maintained in the same way as the BEAS-2B cells. The asthmatics had refractory disease, as defined by the American Thoracic Society [18]. Human lung fibroblasts and smooth muscle cells were obtained by bronchoscopy or from resection tissue from patients undergoing surgery for lung cancer. Fibroblasts and smooth muscle were grown from lung explants, in Dulbecco's modified Eagle's medium (DMEM) (Invitrogen, Paisley, United Kingdom) supplemented with 10% fetal bovine serum (Sigma), penicillin and streptomycin (Sigma), nonessential amino acids, and 110 μg/mL of sodium pyruvate (Invitrogen). All the clinical material was obtained after written informed consent and the study was approved by the Leicestershire Ethics Committee.

### Cell Stimulation

Cells were grown to confluency (which corresponds to approximately 1 × 10^6^ epithelial cells and 200 × 10^3^ fibroblasts or smooth muscle cells) in 6-well plates and then stimulated with either IFN-γ (5 ng/mL), tumor necrosis factor-alpha (TNF-α) (5 ng/mL), interleukin (IL)-4 (10 ng/ml), or a combination of IFN-γ and TNF-α, in 1 mL of fresh growth medium and cultured for 24 hours. The conditioned medium was then aspirated and used for detection of CXCL16 with enzyme-linked immunosorbent assay (ELISA). The cell layers were washed and the cells were either lysed using TRIzol reagent (Invitrogen) for RNA extraction or Laemmli buffer for Western blotting, or detached using nonenzymatic cell dissociation solution (Sigma) for flow cytometry. Primary cells were used at passages 2 to 4.

### Antibodies

Anti-CXCL16 (clone 10B12) and anti-CXCR6 (clone 7F3) were kind gifts from Millenium Pharmaceuticals (Cambridge, MA). Anti-CD3-PE (clone UCHT1), secondary fluorescein isothiocyanate (FITC)-labeled rabbit anti-mouse antibody, goat anti-mouse horseradish peroxidase (HRP)-conjugated antibody, and isotype controls were purchased from (DAKO, Ely, UK). Anti-CXCR6 (clone 56811) was from R&D Systems (Oxford, UK).

### Flow Cytometry

Structural cells were stained with anti-CXCL16 or isotype control antibody at a concentration of 10 μg/mL for 20 minutes. The cells were then washed with phosphate-buffered saline (PBS) with 0.5% bovine serum albumin (BSA), stained with secondary FITC-labeled rabbit anti-mouse antibody for 20 minutes, and washed again before analyzed in a FACSCanto flow cytometer (BD Biosciences, Oxford, UK), using FACSDiva software.

### Chemotaxis Assays

Mononuclear cells were isolated from peripheral blood from a total of 10 healthy volunteers by density gradient centrifugation with Histopaque 1077 (Sigma) and cultured for 7 days in medium (RPMI-1640 supplemented with 10% fetal bovine serum and penicillin and streptomycin) (both Sigma)) with 4 ng/mL of IL-2. The percentage of unstimulated T cells expressing CXCR6 is in the region of 5% [19] and not sufficient to detect a chemotactic response. It was therefore necessary to stimulate the cells with IL-2, which increased the percentage of CXCR6-expressing cells to 19.45% ± 1.76%. Cells were then washed in medium and cell concentration adjusted to 10 × 10^6^ cells/mL. Chemotaxis assays were performed in transwell chambers (5-μm pore size; Fisher Scientific, Loughborough, UK). The lower chamber contained 450 μL of culture supernatants from BEAS-2B cells that had been concentrated 10 times, from 15 to 1.5 mL, using Amicon-Ultra centrifuge devices (Millipore, Watford, UK) with a 10-kDa cutoff. Epithelial cell media was used as a negative control and CXCL12 (R&D Systems) was used as a positive control. One hundred microliters of a cell suspension containing 1 × 10^6^ cells was added to the upper chamber. To block chemotaxis, T cells were either preincubated with 10 μg/mL of anti-CXCR6 monoclonal antibody (clone 7F3), isotype control, or with 100 ng/mL of pertusssis toxin (Sigma). The transwell plate was then incubated at 37°C for 2 hours, and the T cells that had migrated into the lower chamber were counted.

### Western Blotting

Cells were washed once with PBS and lysed in Laemmli buffer (Sigma-Aldrich). Culture supernatants were concentrated 10× using Amicon-Ultra centrifuge devices, before mixed with Laemmli buffer. All protein preparations were boiled for 5 minutes, run on 10% sodium dodecyl sulfate–polyacrylamide gel electrophoresis (SDS-PAGE) gel (100 V). The proteins were then electrotransferred to Immobilion P transfer membranes (Millipore). Membranes were probed with 0.2 μg/mL mouse anti-human CXCL16 or isotype mouse control, followed by 1 μg/mL of goat anti-mouse–HRP antibody. For detection, enhanced chemiluminescence (ECL) reagent (Amersham Biosciences, Little Chalfont, Buckinghamshire, United Kingdom) was used.

### Real-Time RT-PCR

Total RNA was extracted using TRIzol Reagent (Invitrogen) and treated with DNase I (Invitrogen). Reverse transcription (RT) was performed using Omniscript RT kit (Qiagen, Crawley, West Sussex, UK), according to manufacturers protocol.

Real-time polymerase chain reaction (PCR) was performed using Brilliant SYBR Green Master Mix (Stratagene, Amsterdam, The Netherlands) using the following primers: CXCL16: forward: 5′-TCT CAA AGA ATG TGG ACA TGC-3′, reverse 5′-CAG GGG TGT GGA TAT CTG AA-3′, and GAPDH: forward 5′-AAT GGA AAT CCC ATC ACC ATC T-3′, reverse 5′-CGC CCC ACT TGA TTT TGG-3′. Both primers were used at a concentration of 300 nM. The conditions for the PCR were 95°C for 10 minutes followed by 40 cycles of 94°C for 15 seconds, 58°C for 30 seconds, and 72°C for 30 seconds.

Reactions were performed in duplicates and the housekeeping gene GAPDH was used as a normalizer. Fluorescence was measured at the end of each annealing stage and differences were expressed as fold change of the stimulated cells over the unstimulated cells.

### ELISA

Human CXCL16 levels in 24-hour culture supernatants were quantified using Human CXCL16 Quantikine ELISA kit from R&D systems and performed according to manufacturer's protocol. The OD_450_ was read on a Wallac Victor 1420 multilabel counter (Perkin Elmer). The detection limit of the assay was 15 pg/mL.

### Statistical Analysis

Paired two-tailed Student *t* test was used to compare differences and *P* values of less than .05 were considered significant. All statistical analysis was done with Graphpad Prism (Graphpad Software Inc, San Diego, CA).

## RESULTS

### Expression of CXCL16 by BEAS-2B Cells

**FIGURE 1:**
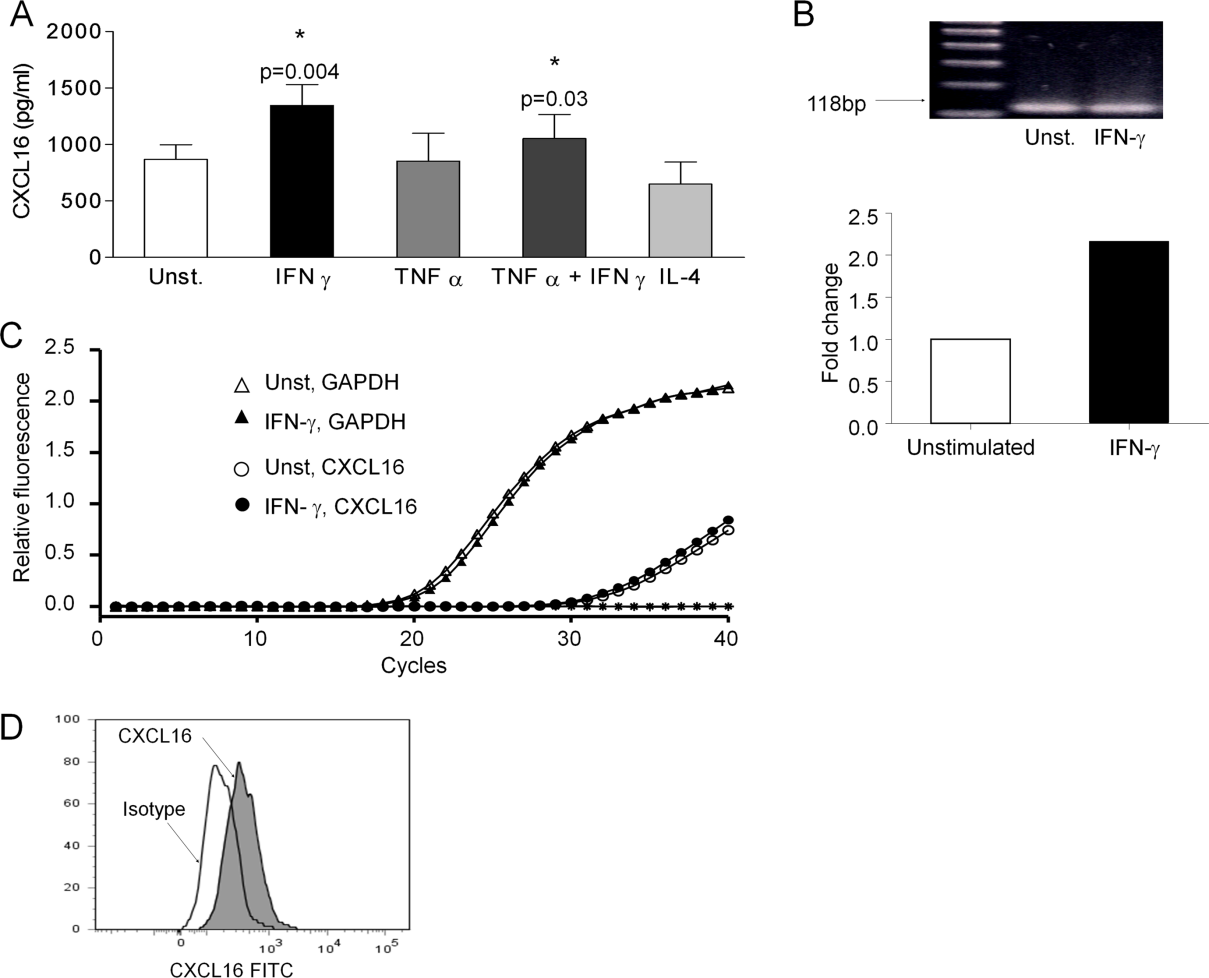
(*A*) Concentrations of CXCL16 in 24-hour culture supernatants from BEAS-2B cells, measured by ELISA. CXCL16 was significantly up-regulated after IFN-γ stimulation (*n* = 6). (*B*) Confirmation by PCR of CXCL16 expression on BEAS-2B cells by the presence of a band at 118 bp. Lane 1 corresponds to 100bp DNA ladder, lane 2 corresponds to unstimulated cells, and lane 3 corresponds to IFN-γ–stimulated cells. (*C*) Real-time PCR amplification plot (*left panel*) and chart (*right panel*) showing a 2.2 times up-regulation of CXCL16 mRNA expression when BEAS-2B cells were stimulated with IFN-γ (*filled symbols*), compared to unstimulated cells (*open symbols*). GAPDH was used as a normalising housekeeping gene (representative of *n* = 2). (*D*) Flow cytometry of BEAS-2B cells detected the membrane-bound form of CXCL16, as demonstrated by a shift in the population when cells had been stained with anti-CXCL16 antibody (*shaded peak*) as compared to isotype control (*blank peak*) (representative of *n* = 5).

Supernatants from BEAS 2B cells (*n* = 6) cultured for 24 hours contained 859.8 ± 128.3 pg/mL of CXCL16 as measured by ELISA. Stimulation with IFN-γ resulted in a significant increase in the release of CXCL16 into the supernatant (1345.0 ± 183.6 pg/ml; *P* = .004, *n* = 6) (Figure 1*A*). No increase in CXCL16 release was seen when cells were cultured with TNF-α or IL-4. mRNA expression of CXCL16 was confirmed using RT-PCR (Figure 1*B*). The effect of IFN-γ stimulation was associated with a 2.2-fold increase in the amounts of CXCL16 mRNA as measured by Real time RT-PCR (*n* = 2) (Figure 1*C*).

The membrane-bound form of CXCL16 was detected on the surface of BEAS-2b cells, using flow cytometry. Figure 1*D* shows an increase in the mean fluorescence intensity when cells had been stained with an anti-CXCL16 antibody (*shaded peak*) compared to the isotype control (*blank peak*) (mean fluorescence intensity increased from 611.6 ± 186.5 to 1694 ± 709.0, 2.64 ± 0.56 times up-regulation; *P* = .046, *n* = 5). There was no detectable increase in expression of the membrane-bound form of CXCL16 after stimulation with IFN-γ (data not shown).

### Primary Structural Lung Cells Express CXCL16

**FIGURE 2:**
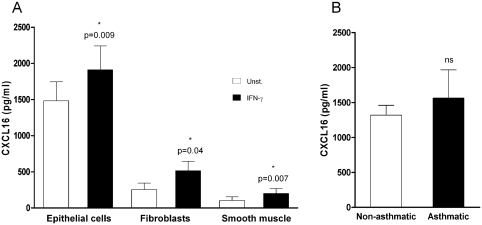
Expression of CXCL16 in primary cultures of human structural lung cells. (*A*) Levels of CXCL16 in 24h culture supernatants of human bronchial epithelial cells (*n* = 6), fibroblasts (*n* = 4), and smooth muscle cells (*n* = 5) were measured by ELISA in unstimulated cultures (*white bars*) and after stimulation with 5 ng/mL of IFN-γ (*black bars*). (*B*) No significant was observed difference between CXCL16 levels measured in primary human bronchial epithelial 24-hour culture supernatants from nonasthmatic (*n* = 3) and asthmatic donors (*n* = 3).

To confirm that CXCL16 expression is a general characteristic of bronchial epithelial lung cells, and not restricted to the BEAS-2B cell line, levels of CXCL16 were measured in supernatants from unstimulated or IFN-γ–stimulated primary cultures of human bronchial epithelial cells. Unstimulated bronchial epithelial cells secreted high concentrations of CXCL16 into the culture supernatants (1483 ± 262 pg/mL; *n* = 6), which were significantly up-regulated after stimulation with IFN-γ (1911 ± 330 pg/mL; *P* = .009, *n* = 6) (Figure 2*A*). CXCL16 has previously been detected at similar concentrations in BAL from both patients with inflammatory lung disease and normal subjects. Consistent with these findings, there was no significant difference between the amounts of CXCL16 produced by bronchial epithelial cultures from normal and asthmatic donors (1319 ± 140.3 pg/mL [*n* = 3], and 1565 ± 401.9 pg/mL [*n* = 3] respectively; *P* = .706) (Figure 2*B*). To investigate the expression of CXCL16 by other structural lung cells, concentrations of CXCL16 in culture supernatants from primary lung fibroblasts and smooth muscle cells were measured. Unstimulated fibroblasts and smooth muscle cells also expressed CXCL16 (253 ± 89 and 103 ± 50 pg/mL, respectively), but at a lower level, which was significantly up-regulated after IFN-γ stimulation (514 ± 132 pg/mL [*P* = .04, *n* = 4] and 200 ± 67 pg/mL [*P* = .007, *n* = 5], respectively) (Figure 2*A*).

### CXCL16 From Epithelial Cell Culture Supernatants Is Expressed as a 35-kDa Protein

**FIGURE 3:**
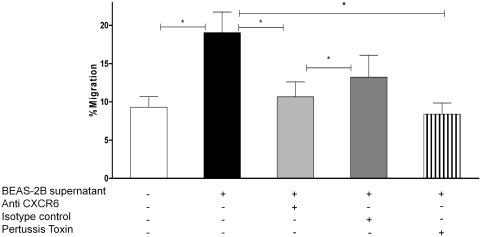
Western blot showing two forms of CXCL16 are produced by BEAS-2B cells. The full-length form of CXCL16 was found in the cell lysates of the BEAS-2B cells, as demonstrated by a strong band at 60 kDa. The truncated form of CXCL16 was mainly detected in the culture supernatants, as a band at 35 kDa (representative of *n* = 3).

Western blotting of cell lysates and culture supernatants from BEAS-2B cells revealed that CXCL16 was expressed as two different forms by these cells: a 60-kDa form that is found predominantly in the cell lysates, and a 35-kDa truncated form that were detected in the supernatants (*n* = 3) (Figure 3). There are several bands at approximately 60 kDa. The basis for this is not known but has been speculated to be due to differences in the extent or type of glycosylation [9].

### Effect of BEAS-2B Supernatants on Migration of T Lymphocytes

**FIGURE 4:**
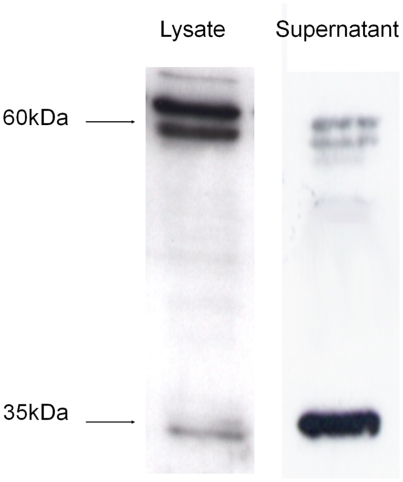
Chemotaxis of peripheral blood mononuclear cells stimulated with IL-2 for 7 days, to 10× concentrated BEAS-2B culture supernatants is expressed as the percentage of cells migrating of the cells added to the upper chamber. Chemotaxis was significantly blocked when T cells were preincubated with anti-CXCR6 (but not with the isotype control) (*n* = 10)), and pertussis toxin (*n* = 3).

After determining that human bronchial epithelial cells produce CXCL16, we assessed the functionality of the secreted protein. Chemotaxis assays were performed, where the ability of BEAS-2B culture supernatants to chemoattract T cells from peripheral blood was tested. Peripheral blood mononuclear cells had been stimulated with IL-2 to up-regulate expression of CXCR6 to 19.45% ± 1.76%. Ten times concentrated BEAS-2B supernatant, containing ∼7 ng/mL of CXCL16, attracted 16.45% ± 2.8% of the cells added to the transwell, and migration was significantly decreased when cells were preincubated with anti-CXCR6 (*P* = .0007, *n* = 10), but not with the isotype control (Figure 4). Pretreatment with pertussis toxin also significantly inhibited migration to 8.39% ± 1.46%, (*P* = .04, *n* = 3), confirming that chemotactic towards the BEAS-2B supernatant was mediated through a G protein–coupled receptor. Human recombinant CXCL12, which was used as a positive-control stimulus, attracted 47.5% ± 6.6% (*n* = 8) of the cells (data not shown); however, two commercially available human recombinant CXCL16 proteins (from R&D systems and Peprotech, used at 100 ng/mL) had no chemotactic activity (data not shown).

## DISCUSSION

This is the first study to investigate generation of CXCL16 by structural lung cells. We have found that CXCL16 is constitutively secreted by bronchial epithelial cells in amounts not usually seen for chemokine secretion without stimulation. This supports the hypothesis that the CXCR6/CXCL16 axis is important in the homeostatic regulation of T-cell migration to the lung.

Chemokines can be divided into those that are constitutively generated and are involved in steady state cell trafficking and immune responses and those that are up-regulated during an inflammatory response [20]. CXCL16 appears to belong to the former category. The substantial baseline expression of CXCL16 that was seen in bronchial epithelial cells was not increased by TNF-α, a cytokine that stimulates the production of most inflammatory related chemokines from structural cells. IFN-γ caused a modest increase in release which may be relevant in the context of Th1 responses, although we have not found that CXCR6 was a Th1-associated chemokine receptor [21]. The constitutive nature of CXCL16 generation is consistent with our previous observation that CXCL16 is found in high concentrations in bronchoalveolar lavage (BAL) but with no difference between normal subjects and those with inflammatory lung disease [7]. Similarly, we found no difference in the amount of CXCL16 released from epithelial cells derived from normal subjects compared to subjects with asthma [7, 21]. The asthmatics were on high-dose inhaled corticosteroids and this could have influenced the results; however, we found no inhibitory effect of dexamethasone on CXCL16 expression by BEAS-2B cells (*n* = 2; data not shown), so we think that is unlikely.

CXCL16, like CXC3L1, is a transmembrane chemokine protein, which is composed of an N terminal chemokine domain, a highly *O*-glycosylated mucin stalk, a single transmembrane helix, and a short cytoplasmic tail. The amino acid sequence suggests a molecular weight of approximately 29 kDa, but glycosylation of the mucin stalk gives a real molecular weight of 60 kDa for the full-length protein.[9, 10] Cleavage of the extracellular domain is thought to be mediated by the disintegrin and metalloprotease ADAM-10 (close to the membrane), yielding a protein of 35 kDa [14, 15]. Western blotting demonstrated that, as might be expected, we were able to detect the full-length form of CXCL16 in epithelial lysates, but the predominant form in the supernatant was the 35-kDa form. In contrast to epithelial cells, we have previously shown and confirmed in this study that the most abundant form of CXCL16 in supernatants from alveolar macrophages is the full-length 60-kDa form rather than the 35-kDa form [7]. Interestingly, we could not detect chemotactic activity in supernatants from alveolar macrophages even though they contained similar amounts of CXCL16 to the epithelial supernatants (data not shown). The reasons for this require further investigation.

Although we were able to detect CXCL16 from all the structural cells we investigated, epithelial cells appeared to be the major producer of this chemokine. This is consistent with the recent observation that CXCL16 is constitutively expressed by keratinocytes, although the concentration of CXCL16 was lower than that seen in the lung [22].

The preferential expression of CXCL16 by epithelial cells suggests that CXCL16 may be involved in the localization of T cells to the epithelium, especially as we have found that CXCR6 is enriched on cells expressing CD103, the receptor for E-cadherin [23].

The CXCL16 in the epithelial supernatant was functional in that the epithelial supernatants contained chemotactic activity for IL-2–stimulated peripheral blood mononuclear cells (PBMCs) (enriched for CXCR6 expression), which was significantly inhibited by an antibody against CXCR6. Similar to Latta and colleagues [24], we were unable to detect any chemotactic activity in 2 commercial sources of recombinant CXCL16. We also found that these proteins failed to stimulate a stimulate calcium signal in a flow cytometry–based calcium flux assay. The reason for this lack of activity is not clear.

It was striking that the blocking monoclonal antibody against CXCR6 was able to inhibit the chemotaxis response completely. This suggests that CXCL16 is responsible for most of the T-cell chemoattractant activity expressed constitutively by bronchial epithelial cells.

In summary, we have found that bronchial epithelial cells constitutively express large amounts of chemotactically active CXCL16. This supports the concept that CXCR6/CXCL16 are important in controlling the homeostatic migration of T cells into the human lung.
